# The psychometric properties of the Persian version of the MUSIC Model of Academic Motivation Inventory among nursing students

**DOI:** 10.1002/nop2.1082

**Published:** 2021-09-28

**Authors:** Mansoureh Ashghali Farahani, Maryam Saeedi

**Affiliations:** ^1^ Nursing Care Research Center Medical‐Surgical Nursing Department School of Nursing and Midwifery Iran University of Medical Science Tehran Iran; ^2^ Saveh University of Medical Sciences Saveh Iran

**Keywords:** motivation, nursing, psychometrics, students, surveys and questionnaires

## Abstract

**Aim:**

To determine the psychometric properties of the Persian version of the MUSIC Inventory in nursing students.

**Design:**

Cross‐sectional psychometric study.

**Methods:**

Transcultural adaptation of the MUSIC Model of Academic Motivation Inventory was carried out using translation to Persian and back‐translation. Then, the qualitative and quantitative face and content validity of the inventory were evaluated. Construct validity was assessed by exploratory factor analysis. To perform construct validity and reliability, a convenience sample of 360 undergraduate nursing students was recruited. Cronbach's alpha was used to assess internal consistency reliability.

**Results:**

Cronbach's alpha for all items of the MUSIC inventory was .94, and each factor was between .72–.93. Exploratory factor analysis supported the 5‐factor structure of the MUSIC inventory. These 5 factors explain 66.59% of the overall extracted variance. Three items of the MUSIC inventory, which were related to the empowerment component, were deleted.

**Conclusion:**

Based on the findings of this study, the Persian version of the MUSIC Model of Academic Motivation Inventory is a valid and reliable tool for Persian language nursing students.

## 1 INTRODUCTION

Motivation is one of the important concepts in nursing education (Miers et al., 2007; Zhang et al., 2015). Providing high‐quality nursing services requires nursing students who are motivated to acquire a wide range of information and skills and a will to continuously learn and re‐learn as the field develops (Bråten & Olaussen, 2007). The results of the literature review showed that the most important factors that have a positive effect on academic motivation in nursing students include the attitude of professors and families towards nursing (Oudi et al., 2006), scientific and practical competencies of instructors in clinical education, instructor behaviour with students (Hanifi et al., 2012), improving the quality of the learning environment (Aktaş & Karabulut, 2016; Karabulut et al., 2015) and a positive attitude towards the courses (Nilsson & Stomberg, 2008).

Instructors play an important role in motivating students (Creţu, 2015). During the educational process and teaching, instructors provide a set of learning opportunities for students that play an important role in promoting academic motivation and academic success and achieve educational goals (Banidavoodi, 2014). Accordingly, Jones (2009) developed the "MUSIC Model of Academic Motivation" to help instructors to design courses that motivate students to learn. Jones's model is designed to create a conceptual framework to help teachers in all disciplines understand how to apply existing research and motivational theories in their course design (Jones & Skaggs, 2016).

The MUSIC Model of Academic Motivation consists of five components that teachers should consider when designing instruction to motivate students. These five components include empowerment, usefulness, success, interest and care, which were derived by Jones from research and theories related to motivation and education. The name of the model (MUSIC) consists of the second letter of “empowerment” and the first letter of the other four components (Jones, 2009).

Based on the MUSIC model, the MUSIC Model of Academic Motivation Inventory was designed by Jones. This inventory could be useful to instructors and researchers interested in assessing the impact of instruction on students' motivational beliefs (Jones & Skaggs, 2016), and also, the MUSIC inventory is a useful tool for teachers and instructors to adjust their educational plans to maximize learning motivation (Pace et al., 2016).

The MUSIC inventory is widely used in the United States for students of different levels and disciplines. The questionnaire was also translated into Arabic (Mohamed et al., 2013), Vietnamese, Icelandic (Schram & Jones, 2016), Spanish and Chinese (Jones et al., 2017), and its validity and reliability were reported in these studies.

The validity and reliability of the MUSIC inventory among medical students have been examined in two studies on pharmacy (Pace et al., 2016) and veterinary students (Jones et al., 2019). There are currently no studies examining the validity and reliability of the MUSIC inventory for measuring academic motivation in nursing students. Therefore, the present study was designed to evaluate the validity and reliability of the MUSIC inventory for nursing students.

### 1.1 Background

Academic motivation is defined as the factors that influence a person to attend school or college (Clark & Schroth, 2010). Motivated students are more willing to engage in learning activities and achieve success in the academic environment. They are more likely to pay attention during academic activities, spend more time for study and use effective learning strategies (Schunk et al., 2008). There is a significant relationship between academic motivation and some academic outcomes such as school grades, effort and persistence at school, and satisfaction with academic activities (Vecchione et al., 2014).

Nursing education, like other disciplines, is based on motivation. Highly motivated nursing students have fewer academic dropouts (Sharififard et al., 2016; Zhang et al., 2015). A positive and significant relationship has been reported between academic motivation and academic self‐esteem in nursing students (Moura, 2006). Also, the motivation of nursing students has shown a positive and significant relationship with their professional commitment (Nesje, 2015).

Given the importance of academic motivation in nursing students, designing and validating scales to measure the academic motivation of nursing students is very important. The MUSIC inventory is a 26‐item self‐report tool that examines students’ beliefs related to the five components of the MUSIC Model of Academic Motivation including empowerment, usefulness, success, interest and care. The empowerment component has five statements that examine students' perceptions of how much they have the power to make decisions and control in the learning process and doing homework. The utility component has five statements that examine students' perceptions of the relevance of course materials to their lives and work. The success component has four statements that measure the level of students' expectations for academic success if they put forth the appropriate effort. The interest component has six statements that examine students' perceptions of how much the instructional methods and coursework are interesting or enjoyable. The care component has six statements that examine students' perceptions of how much a teacher cares about their success in the coursework and their well‐being. For each statement, there is a 6‐point Likert scale ranging from strongly disagree to strongly agree (Jones & Skaggs, 2012).

In research, clinical practice, and health evaluation, measurement scales are critical. The evaluation of instrument psychometrics properties is beneficial in the selection of valid and reliable tools to assure the quality of study results (Souza et al., 2017). Psychometric properties refer to the validity and reliability of the measurement scale (Asunta et al., 2019).

Validity implies how well the information gathered corresponds to the subject of the study (Taherdoost, 2016). A review of the literature reveals that there are various classifications for validity. According to Mokkink et al., validity is classified into 3 types, which include content validity, construct validity and criterion validity. Content validity is defined as “the extent to which an instrument's content accurately reflects the construct being measured” (Mokkink et al., 2010). Construct validity is referred to as “the extent to which data and theory support the test score interpretations implied by proposed test uses” (Embretson, 2007). Criterion validity is defined as “The degree to which an instrument's scores are an accurate reflection of a gold standard.” (Mokkink et al., 2010). Face validity is a type of validity. According to Mokkink et al. Face validity is classified as a subset of content validity. Face validity refers to how relevant the content of a test and its items are to the setting in which the test is administered (Holden, 2010).

Reliability is referred to as “the consistency of an instrument in measuring a certain event”; it assumes that we get the same findings for repeated measurements of the same phenomena (Ursachi et al., 2015). There are several sorts of reliability, including internal consistency, split‐half reliability, test–retest reliability, and homogenous reliability (Ebadi et al., 2017; Mokkink et al., 2010; Ursachi et al., 2015). The Cronbach alpha coefficient is the most widely used internal consistency. When using Likert scales, it is regarded as the most accepted measure of reliability (Taherkhani et al., 2010).

Due to the importance of assessing academic motivation in nursing students and also the importance of localization and determining the psychometric properties of the instrument, including validity and reliability for use in Persian, the present study has been designed. Since the MUSIC inventory has been successfully validated by samples of students of different education levels, we assume that it is also applicable to nursing students. Therefore, our research question is: How valid and reliable is the MUSIC Model of the Academic Motivation Inventory when used with nursing students?

## 2 METHODS

### 2.1 Design

A cross‐sectional preliminary pilot psychometric study was designed. Transcultural adaptation of the MUSIC Model of Academic Motivation Inventory was carried out using translation to Persian and back‐translation. Then, the qualitative and quantitative face and content validity of the inventory were evaluated. Construct validity was performed by exploratory factor analysis. Cronbach's alpha values were used to test the internal consistency reliability of all of the MUSIC Inventory scales. In the following, the psychometric steps are explained in order.

#### 2.1.1 Translation of the instrument

Written permission for the use of the MUSIC inventory was obtained from the developer of the inventory, Professor Brett Jones. The questionnaire was translated from English to Farsi and then back to English using “the Forward & Backward method,” following WHO recommendations. “For forwarding translation, two independent professional translators translated the MUSIC inventory from English into Persian. An expert panel, consisting of two nursing professors as well as two translators, compared and discussed both translations, and the Persian version of the questionnaire was obtained. For back translation, the Persian version of the inventory was translated into English by the translator who had not seen the original version of the inventory and was unaware of the purposes of the study. The panel checked and adapted this version with the original version and prepared the final Persian version” (World Health Organization, 2009).

#### 2.1.2 Content validity

As mentioned earlier, face validity is a form of content validation. In other words, face validity is the first stage of content validity, so we will first describe how to examine face validity.

Face validity was determined by both quantitative and qualitative methods. Qualitative face validity was performed by face‐to‐face interviews with six nursing students from different semesters; they were asked to comment on the ability to understand the inventory items (difficulty level), the relationship of the items to the concept (relevancy) and the presence of incomprehensible words in the items (ambiguity). Participants' comments about the items of the inventory were recorded and reviewed with the research team and the necessary corrections were made in the inventory.

For the quantitative face validity phase, ten nursing students were asked to rate the importance of each item based on the Likert scale of 5 options (quite important = 5, important = 4, almost important = 3, a little important = 2, not important = 1). The impact score of the items was obtained from the following formula: Impact score = Frequency (%) × Importance. An impact score >1.5 was considered appropriate for each item (Ebadi et al., 2017).

To determine qualitative content validity, 10 nursing faculty members and experts in the field of psychology, instrument design and psychometrics were asked to submit their review of the inventory in terms of grammar, wording and item allocation. The recommendation of the experts on the items was carefully examined, and the proposed corrections were applied to the items as much as possible. Quantitative content validity was assessed by measuring the content validity ratio (CVR) and content validity index (CVI). To determine the content validity ratio, 10 nursing faculty members and experts in the field of psychology, instrument design and psychometrics were asked to rate the “essentiality” of the inventory items on a three‐point scale as follows: not essential: 1; useful but not essential: 2; and essential: 3. Then, CVR was calculated based on the Lawshe formula which is acceptable with the score of 0.62 or above (Lawshe, 1975). CVI can be calculated for each item of the scale (item‐level or I‐CVI) and also for all of the items (scale‐level or S‐CVI). To determine the content validity index (CVI), the same ten experts were asked to rate the “relevance” of the MUSIC inventory items on a four‐point scale from 1 to 4. The CVI of each item was computed by dividing the number of experts who had rated that item as 3 or 4 on the total number of experts. For I‐CVI and S‐CVI, a score of 0.9 is considered excellent and 0.8 is acceptable (Ebadi et al., 2017; Polit & Beck, 2006).

#### 2.1.3 Construct validity

Exploratory factor analysis was used to determine the construct validity of the MUSIC inventory. For this purpose, 360 undergraduate nursing students from a nursing school in Iran were selected by convenience sampling method. The nursing students met the following inclusion criteria: (i) willingness to participate in research, (ii) being able to read and write Persian and (iii) no co‐morbid psychiatric problems. Nursing students were excluded if they did not complete the study questionnaires completely. The study questionnaires were a demographic information questionnaire and the MUSIC Model of Academic Motivation Inventory. The MUSIC Inventory was administered two weeks before the end of semester examinations in three specialized nursing courses, medical‐surgical nursing 1, 2, and 3.

Kaiser–Meyer–Olkin (KMO) index was calculated to examine whether the sample size was adequate and a value of 0.8 or more was considered suitable for the factor analysis (Çokluk & Koçak, 2016). Bartlett's test of sphericity was used for the suitability of the factor analysis model. In the next step, after calculating the correlation matrix between the variables, due to the non‐normality of some items, factor extraction was performed using the principal axis factoring (PAF) method and the Promax rotation. At this stage, the variables that have a high correlation with each other are placed in a factor. The factor load of each item in the factor matrix and the rotated matrix was considered at least 0.3 (Kellar & Kelvin, 2013).

Kaiser criterion and scree plot methods were used to determine the number of inventory factors. In Kaiser criterion, any factor with an eigenvalue higher than one is considered as a factor, in scree plot eigenvalues are plotted on the horizontal axis and the number of factors is plotted on the vertical axis, and wherever the graph fails (elbow point), the factors, which are placed before it, is considered as the appropriate factors (Ebadi et al., 2017).

#### 2.1.4 Reliability

The reliability of the MUSIC inventory was evaluated by the internal consistency method. To assess internal consistency, Cronbach's alpha coefficient of data obtained from 360 students in the whole scale and its subscales was calculated. Cronbach's alpha coefficient above .7 is acceptable (Webb et al., 2006). Statistical analyses were performed using SPSS software version 22.

### 2.2 Ethical consideration

This study was approved by the Ethics Committee of Iran University of Medical Sciences (Code: IR.IUMS.REC1395.9221199206). All participants voluntarily participated in this study. Written informed consent was obtained from each participant.

## 3 RESULTS

### 3.1 Face and content validity

The results of face validity showed all items of the inventory were appropriate, clear and important. Table 1 shows the impact scores of the items (to determine quantitative face validity), content validity ratio (CVR) and content validity index (CVI). According to this table, the impact scores of all items were >1.5 and none of the items were removed at this stage.

**TABLE 1:** Impact score, content validity index (CVI) and content validity ratio (CVR) of MUSIC Inventory

Item's no.	Items	CVR score	I‐CVI score	Impact score
1	The coursework held my attention	1	1	1.85
2	I had the opportunity to decide for myself how to meet the course goals	0.8	0.9	2.73
3	In general, the coursework was useful to me	0.8	1	2.4
4	The instructor was available to answer my questions about the coursework	1	1	3.52
5	The coursework was beneficial to me	0.8	1	3.44
6	The instructional methods used in this course held my attention	1	1	4.4
7	I was confident that I can succeed in the coursework	1	0.9	3.2
8	I had the freedom to complete the coursework my own way	1	0.9	2.34
9	I enjoyed the instructional methods used in this course	0.8	1	3.2
10	I felt that I can be successful in meeting the academic challenges in this course	1	1	2.87
11	The instructional methods engaged me in the course	1	1	2.87
12	I had options in how to achieve the goals of the course	0.8	1	2.34
13	I enjoyed completing the coursework	0.8	1	2.22
14	I was capable of getting a high grade in this course	0.4	1	3.28
15	The coursework was interesting to me	0.6	1	2.59
16	The instructor was willing to assist me if I needed help in the course	1	1	3.44
17	I had control over how I learned the course content	0.8	1	3.2
18	Throughout the course, I felt that I could be successful on the coursework	0.8	1	2.8
19	I found the coursework to be relevant to my future	0.8	1	4.14
20	The instructor cared about how well I did in this course	1	1	3.87
21	I will be able to use the knowledge I gained in this course	1	1	4.9
22	The instructor was respectful of me	0.8	1	3.44
23	The knowledge I gained in this course is important for my future	0.8	0.9	4.8
24	The instructor was friendly	0.4	0.9	2.28
25	I believe that the instructor cared about my feelings	1	1	1.75
26	I had flexibility in what I was allowed to do in this course	1	1	3.2
		S‐CVI/Ave		0.976695494
		Total agreement		20
		S‐CVI/UA		0.769230769

Based on the scores obtained from the content validity ratio (CVR), the scores of all items were higher than 0.62. The scores obtained from the content validity index showed that the I‐CVI values of all items were equal to or higher than 0.9 and the S‐CVI value was higher than 0.9 (S‐CVI = 0.97), so at this stage, all items remained in the inventory (Table 1).

### 3.2 Construct validity

According to the inclusion criteria, 360 nursing students from different semesters entered the study and 248 nursing students completed the questionnaires. The response rate was 95.38%. The average age of the participants was 21.17 (*SD* = 4.42) years. The mean scores of students were 16.41(*SD* = 1.41) of 20. The majority of participants were women (58.47%) and were freshmen (37.9%).

The KMO index was 0⋅910 and Bartlett's test was 4,258.700, *p* < .0001 indicating that the sample size is sufficient for factor analysis and the existing matrix can extract factors.

**FIGURE 1:**
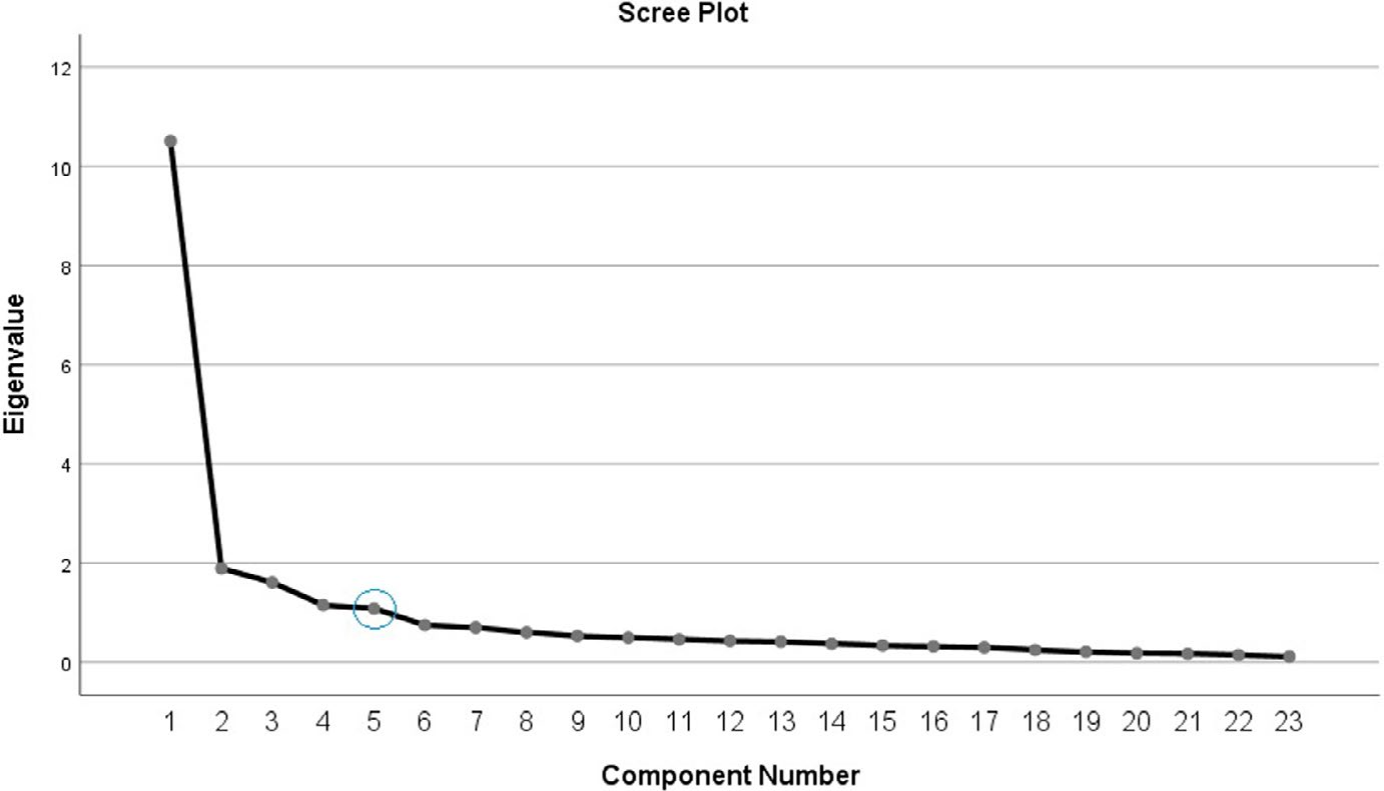
The scree plot of the Persian version of the MUSIC inventory factors

Kaiser criterion indicated six factors in the structure of the MUSIC inventory. As shown in Table 2, the eigenvalues of the six factors were greater than one. Also, in the scree plot, from the sixth factor onwards, the process of graph change is almost homogeneous. However, due to the eigenvalue of factor 6 was slightly more than one and also due to the high cross‐loading of items, factor analysis was performed again by selecting five factors. Besides, as will be noted below, five factors are identified by removing items 2, 8, and, 26 (Figure 1). Based on this, five factors were identified as the main factors of the inventory with eigenvalues of 11.32, 1.94, 1.66, 1.28, and 1.09 respectively, and these factors explained 66.59% of the total common variance (Table 2).

**TABLE 2:** Factor analysis and internal consistency of the MUSIC inventory

Factor name	Items	Factor loading	% of variance	Eigenvalue	Cronbach's alpha
Interest	1. The coursework held my attention	.759	43.555	11.324	.93
	3. In general, the coursework was useful to me	.797			
	5. The coursework was beneficial to me	.645			
	6. The instructional methods used in this course held my attention	.757			
	9. I enjoyed the instructional methods used in this course	.711			
	11. The instructional methods engaged me in the course	.681			
	13. I enjoyed completing the coursework	.623			
	15. The coursework was interesting to me	.723			
Care	4. The instructor was available to answer my questions about the coursework	.716	7.482	1.945	.89
	16. The instructor was willing to assist me if I needed help in the course	.846			
	20. The instructor cared about how well I did in this course	.607			
	22. The instructor was respectful of me	.663			
	24. The instructor was friendly	.767			
	25. I believe that the instructor cared about my feelings	.804			
Success	7. I was confident that I could succeed in the coursework	.720	6.390	1.661	.75
	10. I felt that I could be successful in meeting the academic challenges in this course	.415			
	14. I was capable of getting a high grade in this course	.548			
	18. Throughout the course, I felt that I could be successful in the coursework	.515			
Empowerment	12. I had options on how to achieve the goals of the course	.472	4.953	1.288	.72
	17. I had control over how I learned the course content	.609			
Usefulness	19. I found the coursework to be relevant to my future	.574	4.220	1.097	.77
	21. I will be able to use the knowledge I gained in this course	.490			
	23. The knowledge I gained in this course is important for my future	.643			

The rotated component matrix was used to determine which of the items in the inventory was more correlated with the five factors. Table 2 shows the factor loads after rotation. Based on the results, item 2 was deleted due to the cross‐loading, and items 8 and 26 were deleted due to the lack of factor load on them. Therefore, the Persian version of the MUSIC inventory has a five‐factor structure as follows. The first factor with an eigenvalue of 11.32 consists of eight items including items 1, 3, 5, 6, 9, 11, 13, and 15, which explain 43.55% of the total variance. All items of this factor correspond to the items of interest component in the original inventory except items 3 and 5 which are related to the useful component in the original inventory. The second factor with an eigenvalue of 1.94 consists of six items including items 4, 16, 20, 22, 24, 25, which explained 7.48% of the total variance. All items of this factor correspond to the care component in the original inventory. The third factor with an eigenvalue of 1.66 consists of four items including items 7, 10, 14, and 18, which explained 6.39% of the total variance. All items of this factor correspond to the success component of the original inventory. The fourth factor with an eigenvalue of 1.28 consists of two items including items, 12 and 17, which explained 4.95% of the total variance. The items of this factor correspond to the empowerment component in the original inventory. The fifth factor with an eigenvalue of 1.097 consists of three items including items 19, 21, 23, which explains 4.22% of the total variance. The items of this factor correspond to the usefulness component in the original inventory.

### 3.3 Reliability

The results of evaluating the reliability of the Persian version of the MUSIC inventory by internal consistency method showed that Cronbach's alpha coefficient for the whole inventory, after deleting three items, was .94 and for each of its components was between .72 and .91 (Table 2).

## 4 DISCUSSION

According to the present study, the Persian version of the MUSIC Model of Academic Motivation Inventory is potentially a valid and reliable tool for Persian language nursing students.

In the present study, the Cronbach's alpha coefficient for the whole inventory was .94 and for each of its components was between .72 and .93. Considering that Cronbach's alpha coefficient is above .7 is acceptable (Webb et al., 2006), therefore, the results indicate that the internal consistency of the Persian version of the MUSIC inventory is desirable. Consistent with this finding, the results of the study of Pace et al. (2016) also showed that Cronbach's alpha coefficient for each of the components of the inventory was close to or above .9, and in similar studies on the psychometrics of the MUSIC inventory, appropriate Cronbach's alpha and acceptable reliability have been reported (Jones et al., 2019; Jones & Skaggs, 2012, 2016; Mohamed et al., 2013; Pace et al., 2016).

In the present study, Cronbach's alpha for the empowerment components is less than other components (α = .72). It could be due to the deletion of items in this component and reducing the number of items. In the present study, Cronbach's alpha was the highest for the interest component (α = .93). The results of the present study are in contrast to the results of the study of Jones et al. (2019), in which the lowest Cronbach's alpha was for the interest component (α = .78) and the highest was for the utility component (α = .93). This can be due to the variability of the number of items in each of these components.

In this study, exploratory factor analysis supported the 5‐factor structure of the MUSIC inventory. These 5 factors explain 66.59% of the overall extracted variance. Two‐factor structures have been proposed for the MUSIC model: the seven‐factor structure and the five‐factor structure. The seven‐factor model consists of the factors of empowerment, usefulness, success, situational interest, personal interest, academic care and personal care. While the five‐factor model is composed of the factors of empowerment, usefulness, success, interest and care (Jones & Wilkins, 2013). Although both structures of the MUSIC model are credible representatives of this model, the study of Jones and Wilkins (2013) showed that the five‐factor structure had a stronger theoretical basis than the seven‐factor structure.

In the present study, the results of exploratory factor analysis initially determined the presence of six factors in the inventory, which by removing the three items 2, 8, and 26 and re‐performing factor analysis, five factors were identified. Consistent with the findings of this study, in the study of Pace et al. (2016) exploratory factor analysis showed five distinct factors in the MUSIC inventory. In the study of Parkes et al. (2017), the results of confirmatory factor analysis, while confirming the five structural factors of the MUSIC model, showed that the five factors of the MUSIC model are separate and students can understand these five factors differently. These findings are comparable to the findings of Mikaeli et al. (2017) in which it was reported that the seven‐factor model has a better fit than the five‐factor model. Jones et al. (2019) in their study of MUSIC inventory psychometrics in veterinary students, based on the results of confirmatory factor analysis, concluded that the data fit well with the five‐factor structure of the MUSIC model.

In this study, some items of inventory were changed during the validity process. Items 2, 8, and 26 were also removed. All three items are related to the empowerment component. The empowerment component examines students' perceptions of the extent to which they have decision‐making and controlling power in learning and doing homework (Jones, 2009). The construct associated with empowerment is autonomy. Autonomy as a psychological need is an essential element for learning motivation (Rose, 2011). By empowering students, educators can help meet their need for autonomy and empower them by providing them with the ability to make decisions and choices (Jones et al., 2016). The degree of choice and control that a teacher gives to the students in learning experiences can be significantly effective in intrinsic interest and motivation because classroom choice personalizes learning and makes it important to the students (Valerio, 2012). Students with lower self‐regulation are less attentive to academic issues and have more academic failure (Mohammadreza et al., 2014).

The most important reasons for removing these three items (2, 8, and 26) in this study may be related to differences in teaching methods and the educational systems in the context of research in which does not give the student freedom in selecting learning objectives and assignments and the student has to do homework according to the tasks predetermined by the instructor or the faculty and according to the syllabus, regardless of whether it is to his liking or not. To empower nursing students, educational activities and teaching methods should be designed to foster creative and critical thinking in nursing students so that they can apply their knowledge in clinical situations (Mohsenpour & Vanaki, 2005).

The results of factor analysis showed that items 3 and 5 are more correlated with the interest component but, according to the purpose of the items, are related to the usefulness component. One of the reasons could be that students are more interested in learning content that is useful and practical for them (Najafi Kalyani et al., 2011), and these two components (interest and usefulness) may have overlapped. The MUSIC factors were moderately correlated with each other. For example, in the study by Jones et al. (2019), the correlation between usefulness and interest components was .79. One study found that telling students that a task is useful increases their interest in it, but only if they have a higher understanding of task‐related success (Durik et al., 2015). One of the main challenges in nursing education is the gap between theory and clinical practice (Aliafsari Mammaghani & Zamanzadeh, 2017). Determining the application of theoretical content in the clinic reduces the distance between theory and clinical practice and increases students' interest in the content (Kermansaravi et al., 2013).

Consistent with the findings of this study, Jones and Skaggs (2016) in a study aimed at determining the validity of the MUSIC inventory in students of different disciplines showed that the MUSIC inventory is a valid and reliable tool and can be used by professors and researchers to evaluate the effect of instruction on students' motivational beliefs. Pace et al. (2016) in their study reported that the MUSIC inventory has sufficient reliability and validity in pharmacy students and it can be a useful tool for pharmacy faculty members to adjust their instructional plans to create maximum learning motivation in students. The results of the study by Parkes et al. (2017) also showed that the MUSIC questionnaire can be used as a valid and reliable tool for measuring the academic motivation of music students (Parkes et al., 2017).

### 4.1 Implications for nursing education and research

In this study, the Persian version of MUSIC inventory was prepared for nursing students and its validity and reliability were evaluated. Nursing instructors can use this inventory to assess their educational strategies and use the scores obtained from this inventory to examine the strengths and weaknesses of their instruction and to improve their instruction and motivate students. By assessing students' perceptions of MUSIC components, the instructors can work on components that have earned them fewer points. For example, if the usefulness component scores are low, the instructor should emphasize more on the application of instructional content and link the instructional content to clinical situations. At the programme and faculty level, the use of this inventory can determine students' perceptions about the strengths and weaknesses of curricula and teaching strategies in different courses. In addition, this questionnaire can be used for research purposes in the field of nursing education to assess students' perceptions of teaching and to evaluate teaching motivational strategies.

### 4.2 Limitations

There were some limitations in the present study. First, recruiting a convenience sample of nursing students from one nursing faculty may limit the generalizability of the findings to Persian nursing students. Second, other suggested psychometric characteristics including convergent, concurrent and discriminant validity were not tested. Moreover, there are other countries besides Iran that speak Persian; the use of this questionnaire in these countries should be adapted according to their culture.

## 5 CONCLUSION

Based on the findings of this study, the Persian version of the MUSIC Model of Academic Motivation Inventory is a valid and reliable tool for Persian language nursing students.

## CONFLICT OF INTERESTS

The authors declare that they have no conflicts of interest.

## AUTHOR CONTRIBUTIONS

Study design: MS and MAF. Data gathering and sampling: MS. Data analysis: MS and MAF. Initial manuscript draft: MS. Critical revision of the manuscript: MAF.

## Data Availability

The data that support the findings of this study are available from the corresponding author upon reasonable request.
